# Caterpillars selected for large body size and short development time are more susceptible to oxygen-related stress

**DOI:** 10.1002/ece3.551

**Published:** 2013-04-08

**Authors:** Jon F Harrison, Arianne J Cease, John M VandenBrooks, Todd Albert, Goggy Davidowitz

**Affiliations:** 1 School of Life Sciences, Arizona State UniversityTempe, Arizona, 85287-4501; 2 School of Biological Sciences, University of SydneySydney, Australia, 2006; 3 Department of Entomology, University of ArizonaTucson, Arizona, 85721-0036

**Keywords:** Growth, hyperoxia, hypoxia, *Manduca sexta*, selection, size

## Abstract

Recent studies suggest that higher growth rates may be associated with reduced capacities for stress tolerance and increased accumulated damage due to reactive oxygen species. We tested the response of *Manduca sexta* (Sphingidae) lines selected for large or small body size and short development time to hypoxia (10 kPa) and hyperoxia (25, 33, and 40 kPa); both hypoxia and hyperoxia reduce reproduction and oxygen levels over 33 kPa have been shown to increase oxidative damage in insects. Under normoxic (21 kPa) conditions, individuals from the large-selected (big-fast) line were larger and had faster growth rates, slightly longer developmental times, and reduced survival rates compared to individuals from a line selected for small size (small-fast) or an unselected control line. Individuals from the big-fast line exhibited greater negative responses to hyperoxia with greater reductions in juvenile and adult mass, growth rate, and survival than the other two lines. Hypoxia generally negatively affected survival and growth/size, but the lines responded similarly. These results are mostly consistent with the hypothesis that simultaneous acquisition of large body sizes and short development times leads to reduced capacities for coping with stressful conditions including oxidative damage. This result is of particular importance in that natural selection tends to decrease development time and increase body size.

## Introduction

**Figure 1:**
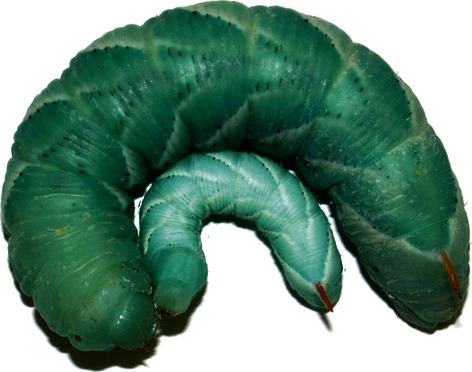
An early and late fifth instar of *Manduca sexta*, the tobacco hornworm. Over 90% of the entire mass gain during development occurs in the final instar (photo credit: Goggy Davidowitz).

In most species, fitness and size are positively correlated (Dmitriew 2011). Larger individuals tend to have higher fecundity (Brown and Mauer 1986; but see Davidowitz 2008). Field studies have detected substantial evidence for directional selection for large body size (Kingsolver and Pfennig 2004; Kingsolver and Diamond 2011) and short development time (Kingsolver and Huey 2008). Nonetheless, there remains substantial variation in body size among individuals, and the mean size of most animals is small, suggesting countervailing selection for smaller sizes, or constraints on achieving large size. A variety of ecological and behavioral mechanisms that select against large animal size have been demonstrated, including greater energy costs, space and nutrient requirements, and increased risks of predation or weather-related injury (Gould 1966; Blanckenhorn 2000; Kozlowski and Gawelczyk 2002; Woodward and Hildrew 2002; Allen et al. 2006; Gotthard et al. 2007; Roy 2008). Many of the ecological costs of achieving large size relate to increased size being associated with longer development times, suggesting that there should be strong selection for higher growth rates. However, recent studies show a large degree of variation in growth rates within animal populations, and that growth rates are often submaximal even in the absence of predation, suggesting that achieving high growth rates requires trade-offs due to allocation away from other functions (Dmitriew 2011). Higher growth rates in animals could have costs in terms of trade-offs with maintenance capacities and/or the ability to resist stressors such as disease, plant allelochemicals, reactive oxygen species, or environmental variation. To test this idea, we exposed populations of the tobacco hornworm, *Manduca sexta* (Sphingidae, Fig. 1), that were artificially selected simultaneously for either large size and short development time or small size and short development time to the respiratory stress of varying atmospheric oxygen levels. We ask the question, “Are caterpillars selected for large size and short development times more sensitive to environmental stress?”

Selection for large size or high growth rate can lead to trade-offs associated with decreased performance. Growth consumes a large portion of an organism's energy and nutrient intake (Wieser 1994; Peterson et al. 1999), and accelerating it must come at a cost to other body functions (Dmitriew 2011). For instance, *Drosophila melanogaster* selected for large size has been shown to have reduced longevity, later-life fecundity (Hillesheim and Stearns 1992), and reduced juvenile viability (Partridge and Fowler 1993). Similar trade-offs are observed in populations exhibiting natural variation in growth rate or size. Faster-growing common frogs from high latitudes show greater predation-independent mortality than lower latitude frogs (Laurila et al. 2008) and butterfly populations with high intrinsic rates of growth are more sensitive to starvation (Gotthard et al. 1994). The mechanisms responsible for such trade-offs remain unclear, but it has been suggested that they include lower energy reserves or reduced levels of proteins involved in repair and maintenance in faster-growing organisms (Dmitriew 2011).

Many laboratory rearing experiments are performed under what researchers hope are optimal conditions, but discovery of trade-offs associated with increased growth rates are most likely when conditions are suboptimal (Fisher et al. 2007; Dmitriew 2011). Beetles selected for large size suffered a greater reduction in body mass when reared under stressful conditions such as being fed on small seeds or reared at high larval density (Amarillo-Suarez et al. 2011). Yellow dung flies selected for large size grew fast on optimal diets, but showed more strongly reduced growth and greater mortality than control lines or lines selected for small sizes when reared under food-restricted conditions, supporting the idea that stressful conditions reveal costs of large size/high growth rates (Teuschl et al. 2006; Blanckenhorn et al. 2011). These studies support the hypothesis that animals selected for large size and short development times might have reduced energy reserves, but do not explicitly address the question of whether such animals have reduced capacities to cope with stresses other than nutrient limitation.

Increases or decreases in atmospheric oxygen level can be a powerful stressor on insect survival and productivity (Charette et al. 2011). While terrestrial insects such as *M. sexta* do not typically experience ambient hypoxia (po_2_ <21 kPa) or hyperoxia (po_2_ >21 kPa) during their normal lifespans, over geologic timescales, insects have experienced considerable variation in atmospheric po_2_ (Harrison et al. 2010). Both hypoxic and hyperoxic conditions can lead to the production of reactive oxygen species (Turrens 2003), which are believed to be chronically produced in all animals, and have been modeled as explicit costs of higher growth rates (Mangel and Munch 2005). In *Drosophila*, the level of hypoxia used here (10 kPa) did not increase oxidative damage as indexed by protein carbonyl production rates (Rascón and Harrison 2010); however, hyperoxic treatments of above 33 kPa have been shown to induce oxidative brain damage (Kloek et al. 1978). Hypoxia may also be generally important during development of insects as recent studies have shown that in *M. sexta*, metabolic rates cease increasing with mass after caterpillars surpass the critical weight, suggesting that metabolism later in the instar may be oxygen-limited, constraining growth, and inducing molting (Callier and Nijhout 2011). Caterpillar pupae may also experience hypoxia during flooding as they are buried in the ground. Studies with a variety of insects have shown that hypoxia generally reduces insect body size and growth rates (Peck and Maddrell 2005; Harrison et al. 2009, 2010; Harrison and Haddad 2011; Heinrich et al. 2011; VandenBrooks et al. 2012). The mechanisms for the negative effects of hypoxia on growth rate and size remain unclear and may vary with life stage and species. Hypoxic-induced reduction in growth rate can occur without an effect on metabolic rate, at least in *D. melanogaster* (Klok et al. 2010). Hypoxia may also induce a reduction in the critical weight, which is the body size at which *M. sexta* initiates the endocrine processes that lead to molting (Callier and Nijhout 2011). Hypoxia can reduce feeding rates of *D. melanogaster*, potentially leading to lower growth rates by reducing nutritional intake (Frazier 2007), and can reduce cell size (Heinrich et al. 2011). Severe hypoxia (2 kPa oxygen) strongly reduces longevity and is associated with greater rates of accumulation of oxidative damage (Rascón and Harrison 2010).

While ambient hyperoxia is even less ecologically relevant to extant insects, insects have evolved through periods of significant hyperoxia (Harrison et al. 2010). Also, hyperoxia provides a convenient experimental manipulation to induce oxidative damage, which is also known to occur during aging or in association with ingestion of toxic plant allelochemicals. The effects of hyperoxia on growth and size in insects are more variable than that have been reported for hypoxia. In *D. melanogaster* exposed to multiple generations of hyperoxia, body size increases (Harrison and Haddad 2011); in single-generation studies, there is no significant effect on size, development time, or growth rate up to a po_2_ of 40 kPa (Klok et al. 2009). At higher levels of hyperoxia, size and survival strongly decrease in *D. melanogaster*, and hyperoxic exposure is associated with damage by reactive oxygen species (Philott et al. 1974; Kloek et al. 1978; Walker and Benzer 2004; Rascón and Harrison 2010). In the cockroach, *Blatella germanica*, growth rate, development time, and survival are negatively affected by both hypoxia and hyperoxia; body size is reduced by hypoxia and weakly or not affected by hyperoxia (VandenBrooks et al. 2012). It has previously been shown that in *M. sexta*, hyperoxia (po_2_ 40 kPa) does not affect the maximal larval mass (Callier and Nijhout 2011). In sum, these data suggest that although hyperoxia can occasionally provide benefits, it is more commonly neutral or stressful for insects, most likely due to increased reactive oxygen species production and subsequent tissue damage. Thus, we predicted that *M. sexta* selected for large body size and short development time would exhibit greater negative responses to variation in oxygen rearing levels, especially hyperoxic conditions known to increase oxidative stress, compared to unselected control lines or lines simultaneously selected for small body size and short development time.

## Materials and Methods

### Genetic selection of *M. sexta*

From a common outbred population of *M. sexta* (described in Davidowitz et al. 2012), three selection lines were established at the University of Arizona in which larvae were simultaneously selected for large body size and short development time (big-fast line), for small body size and short development time (small-fast line), or selected at random in the control line. This experiment was repeated with three independent selection lines at Duke University; responses were very similar for the two independent selection experiments (G. Davidowitz et al., unpubl. ms.). Body size was measured as pupal mass and development time as the number of days from hatching to the onset of wandering (the initial phase of pupation; Davidowitz et al. 2012). We selected 10 generations at 25% simultaneous truncation selection from approximately 240 individuals each generation. For example, in the big-fast line, the 25% largest and 25% fastest individuals were selected as parents for the next generation. Males and females were selected separately to account for sexual size dimorphism (Stillwell and Davidowitz 2010). After 10 generations of selection, the lines were kept at a reduced selection pressure of 50% simultaneous truncation selection for another 10 generations using the same method as described for the 25% simultaneous selection. The larvae used in the oxygen rearing study were derived from these later generations of the lines selected in Arizona, and thus had experienced more than 20 generations of selection.

### Heritabilities and variances in selected lines

We estimated realized narrow-sense heritabilities (*h*^2^) of the big-fast and small-fast lines of the University of Arizona populations in the last two generations (9 and 10) of the 25% truncation selection regime for both body size (pupal mass) and development time (days from hatching to wandering) (Davidowitz et al. 2012) using the breeders equation *h*^2^ = *R/S* where *R* is the response to selection and *S* is the selection differential. It was not possible to calculate realized heritabilities for the control line as this line was selected at random.

### Oxygen rearing experiment

Approximately 250 eggs from 120 adult pairs from each of the three different lines of *M. sexta* (big-fast line, small-fast line, and control) were placed on *M. sexta* medium (Davidowitz et al. 2003) and allowed to hatch. Within 24 h of hatching, 105 larvae from each line were randomly selected and placed individually into separate clear plastic 177-mL containers and fed a standard artificial diet (Davidowitz et al. 2003). The containers were then placed into five different Plexiglas® oxygen-controlled rearing chambers (10, 21, 25, 33, and 40 kPa oxygen). The partial pressure of oxygen was regulated and recorded with a Sable Systems® (Sable Systems, Inc., Las Vegas, NV) ROXY-8 oxygen regulation system. All chambers were housed in VWR® (VWR International, Radnor, PA) incubators maintained at 25°C and approaching 100% relative humidity.

The individual larvae were monitored daily and the food provided ad libitum. Once the larvae reached a mass greater than 3 g, they were transferred to larger 354-mL containers and again food was provided ad libitum. Larvae were weighed on a Mettler® (Mettler-Toledo LLC, Columbus, OH) AE240 digital scale daily. Upon reaching the wandering stage, the larvae were transferred into cups containing aspen shavings and no food to allow them to prepare for pupation. After pupation, each pupa was placed into a glass vial capped with a cotton plug in order to allow equilibration with the treatment oxygen atmosphere during pupal development, while ensuring that adult moths could not escape from the vials after eclosion.

### Measurements of *M. sexta* performance during the oxygen rearing experiment

Growth rates were calculated by subtracting initial larval mass from maximal larval mass and dividing by the number of days between hatching and maximal larval mass. Maximal larval body mass was defined as the highest measured larval mass; this usually occurred a day or two before wandering and pupation. Larval development time was defined as the number of days from hatching to maximal larval mass. Adult mass was measured within 24 h of eclosion. Using a Mitutoyo® (Mitutoyo America, Aurora, IL) (CD-6″BS) digital caliper, we measured adult body length as the distance from the tip of the abdomen to the point where the proboscis enters the head. We analyzed survival by comparing the proportion of animals that survived from egg through eclosion to adult in each treatment group.

### Statistical analyses for oxygen rearing experiment

Prior to analyses, all data were checked for the assumptions of parametric tests. Individual *M. sexta* values for maximal larval mass and adult mass were natural log-transformed to meet assumptions of equal variance. We defined data points as outliers if they had an absolute studentized deleted residual >4 for any variable and a Cook's distance >4/(*n* − *k* − 1), where *n* is the number of cases and *k* is the number of independent variables. Five of the 280 cases were considered outliers and not included in these analyses. For all analyses with the exception of survival rate, we considered only individuals that survived to adulthood. We tested for heteroscedasticity in the lines, and in their response to oxygen, using Levene's test.

The effects of line selection and oxygen level on growth rate, development time, maximum larval mass, adult body length, and adult body mass for all individuals were generally tested using a multivariate analysis of variance (MANOVA), followed by univariate two-way analyses of variance (ANOVAs) and Dunnett's tests. Data for hypoxia (10 and 21 kPa po_2_) were analyzed separately from hyperoxia (21, 25, 33, and 40 kPa po_2_) because we considered these fundamentally different types of stress. Dunnett's test is designed to identify groups whose means are significantly different from the mean of a reference group. We used this procedure to test our a priori null hypothesis that individual groups reared in either hypoxic or hyperoxic conditions will not differ from the reference group reared in normoxia within each line. To compare differences among lines at any given po_2_ (i.e., control, small-fast line, and big-fast line reared at normoxia), we used ANOVAs followed by Tukey HSD tests.

In three cases, Levene's tests indicated significant heteroscedasticity that was not eliminated by transformations: growth rate in hypoxia, and development time and adult body length in hyperoxia (Table 1). For these cases, we used weighted ANOVA's to eliminate heteroscedasticity (Neter and Wasserman 1974). Each observation was weighted by the inverse of the variance of the residuals taken from the respective original line × O_2_ two-way ANOVAs.

**Table 1:** Multivariate analysis of variance (MANOVA) table

MANOVA	Source	Effect df	Error df	Wilks	*F*	*P*
Hypoxia	Model	5	77	0.98	0.26	0.94
	Line	10	154	0.37	10.03	<0.0001
	O_2_	5	77	0.60	10.41	<0.0001
	Line × O_2_	10	154	0.95	0.40	0.94
Hyperoxia	Model	5	202	0.99	0.61	0.70
	Line	10	404	0.51	16.33	<0.0001
	O_2_	15	558	0.54	9.16	<0.0001
	Line × O_2_	30	810	0.64	3.16	<0.0001

We included five response variables: maximum larval mass, development time, growth rate, adult body length, and adult mass that were transformed to *Z*-scores prior to analysis.

We used a maximum likelihood chi-square analysis to test for differences in proportion of *M. sexta* surviving to adulthood. Main effect tests were followed by a priori planned comparisons in hyperoxia to determine whether oxygen rearing level had a within-line effect on survival. Between-line differences at normoxia were tested using chi-square analyses with a modified false discovery rate procedure for multiple comparisons to control for experiment-wise Type I and Type II errors (Benjamini and Yekutieli 2001), setting *α* = 0.027 for between-line multiple comparison.

Throughout, unless otherwise stated, statistical significance was judged as *α* <0.05. In all the figures, planned comparison differences within lines are designated by asterisks and post hoc differences between lines at normoxia are designated by lower case letters. All statistical analyses were performed using Statistica® (Statsoft Inc., Tulsa, OK) 9, 10, or JMP 9.0.0 (SAS, Carey, NC).

## Results

### Realized narrow-sense heritabilities and variances in the selected lines

After 10 generations of selection, genetic variation for both pupal mass and development time was still evident in both selection lines: big-fast *h*^2^_pupa_ = 0.24 and *h*^2^_development time_ = −0.23; small-fast *h*^2^_pupa_ = 0.48 and *h*^2^_development time_ = −0.55.

### Differences in growth, development, and survival among the selected lines in normoxia

**Figure 2:**
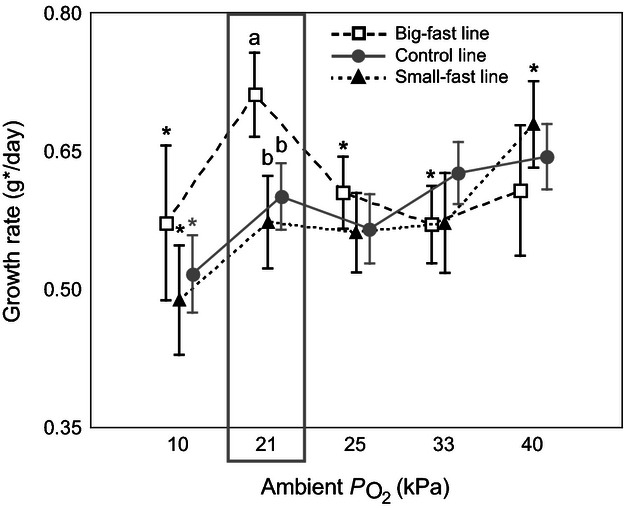
Effects of varying oxygen levels on growth rate. In this and Figures 5, asterisks indicate within-line differences from normoxia (21 kPa po_2_, as indicated by the gray rectangle), determined by a priori planned comparison tests. Letters indicate significant differences among the lines in normoxia, tested with Tukey post hoc test. Vertical bars denote 95% confidence intervals.

**Figure 3:**
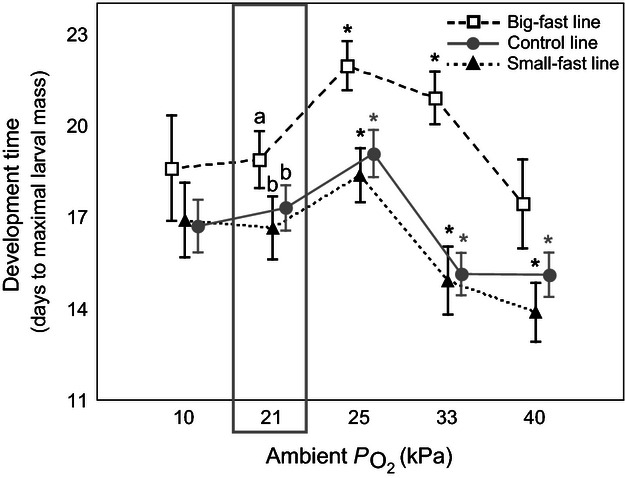
Effects of varying oxygen levels on development time.

**Figure 4:**
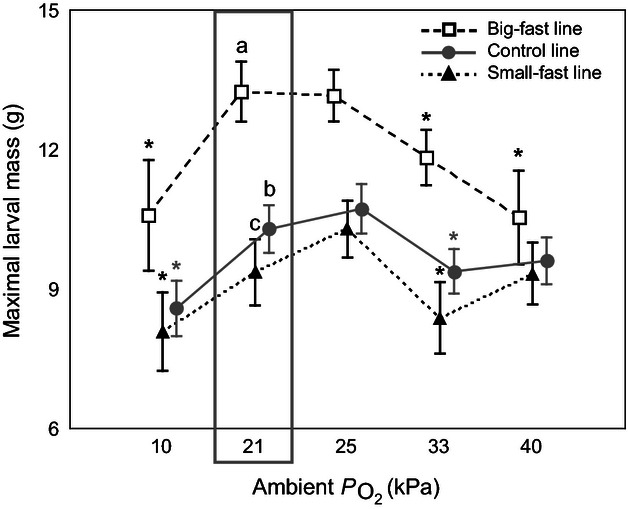
Effects of varying oxygen levels on maximal larval mass.

**Figure 5:**
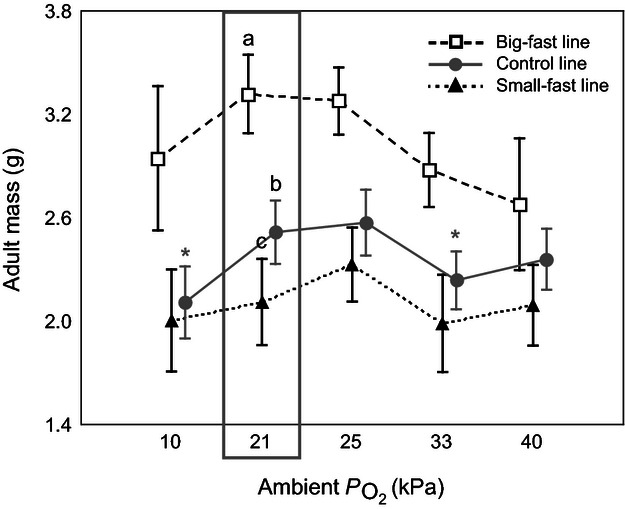
Effects of varying oxygen levels on adult mass.

**Figure 6:**
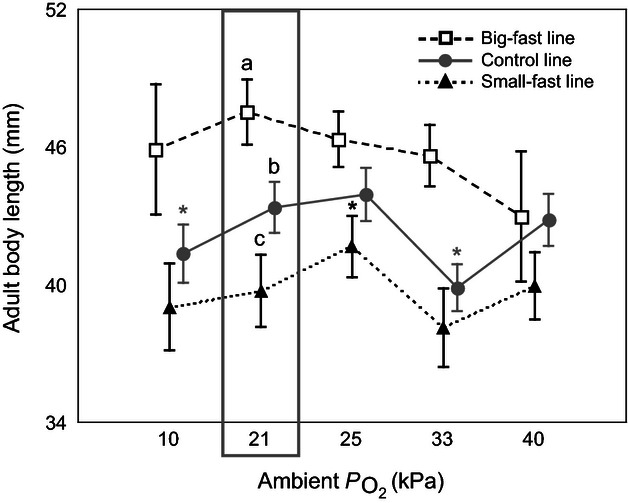
Effects of varying oxygen levels on adult body length.

**Figure 7:**
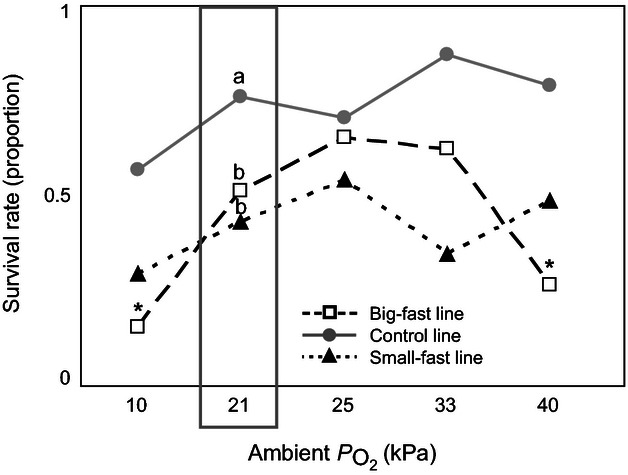
Effects of varying oxygen levels on survival rate (proportion surviving to adult).

Big-fast selected caterpillars had faster growth rates, longer development times, greater maximal larval and adult masses, and longer adults than the control or small-fast selected lines (Figs. 2–6, Tables 1–3). The small-fast selected caterpillars had significantly smaller maximal larval and adult masses, and shorter adults than control lines, but did not differ significantly from the control lines in growth rates or development times. Both the big-fast and small-fast selected lines had a significantly reduced proportion of caterpillars surviving relative to control, randomly selected lines (Fig. 7, Table 4).

**Table 2:** Hypoxia analysis of variance (ANOVA) table

Response variable	Source	df	SS	MS	*F*	*P*
Growth rate	Model	1	77,270.80	77,270.8	77,652.61	<0.0001
	Line	2	388.94	194.47	195.43	<0.0001
	O_2_	1	612.82	612.82	615.85	<0.001
	Line × O_2_	2	16.03	8.01	8.05	<0.001
	Error	87	86.57	1.00		
Development time	Model	1	21,321	4974	4974	<0.0001
	Line	2	42.6	21.3	5.0	0.009
	O_2_	1	0.45	0.75	0.17	0.68
	Line × O_2_	2	2.82	1.4	0.33	0.72
	Error	87	372.9	4.29		
Maximal larval mass	Model	1	363.39	363.39	26991.73	<0.0001
	Line	2	0.90	0.45	33.26	<0.0001
	O_2_	1	0.60	0.60	44.89	<0.0001
	Line × O_2_	2	0.02	<0.01	0.62	0.54
	Error	87	1.17	0.01		
Adult mass	Model	1	54.51	54.51	2158.30	<0.0001
	Line	2	1.58	0.79	31.22	<0.0001
	O_2_	1	0.24	0.24	9.32	0.003
	Line × O_2_	2	0.06	0.03	1.20	0.31
	Error	86	2.17	0.03		
Adult body length	Model	1	112,062.7	112,062	15,917.38	<0.0001
	Line	2	430.4	215.2	30.57	<0.0001
	O_2_	1	31.9	31.9	4.54	0.04
	Line × O_2_	2	6.3	3.1	0.45	0.64
	Error	82	577.3	7.0		

MS, mean square; SS, sum of squares.

**Table 3:** Hyperoxia analysis of variance (ANOVA) table

Response variable	Source	df	SS	MS	*F*	*P*
Growth rate	Model	1	75.3	75.3	7815	<0.0001
	Line	2	0.02	0.02	1.1	0.34
	O_2_	3	0.15	0.05	5.1	0.002
	Line × O_2_	6	0.24	0.04	4.2	<0.001
	Error	227	2.19	<0.01		
Development time	Model	1	312,760.0	312,760	363,169.2	<0.0001
	Line	2	3113.3	1556.7	1807.6	<0.0001
	O_2_	3	4105.8	1368.6	1589.2	<0.0001
	Line × O_2_	6	1570.6	261.8	304.0	<0.0001
	Error	227	195.5	0.9		
Maximal larval mass	Model	1	1105.4	1105.45	61,995.94	<0.0001
	Line	2	2.14	1.071	60.06	<0.0001
	O_2_	3	0.96	0.320	17.93	<0.0001
	Line × O_2_	6	0.21	0.035	1.98	0.07
	Error	227	4.05	0.018		
Adult mass	Model	1	155.89	155.89	4586.308	<0.0001
	Line	2	3.21	1.60	47.281	<0.0001
	O_2_	3	0.86	0.29	8.452	<0.0001
	Line × O_2_	6	0.12	0.02	0.597	0.73
	Error	222	7.55	0.03		
Adult body length	Model	1	141,243.5	141,243	139,745.9	<0.0001
	Line	2	986.2	493.1	487.9	<0.0001
	O_2_	3	588.4	196.1	194.0	<0.0001
	Line × O_2_	6	441.0	73.5	72.7	<0.0001
	Error	211	213.3	1.0		

MS, mean square; SS, sum of squares.

**Table 4:** Maximum likelihood chi-square analysis of survival to adult

	Main effects	Within-line effects of O_2_	df	Chi-square	*P*
Hypoxia	Line		2	14.72	<0.001
	O_2_		1	10.99	<0.001
	Line × O_2_		2	2.56	0.28
		Big-fast	1	10.94	<0.001
		Small-fast	1	1.56	0.21
		Control	1	3.17	0.07
Hyperoxia	Line		2	9.19	0.01
	O_2_		3	13.96	0.003
	Line × O_2_		6	16.00	0.01
		Big-fast	3	13.96	0.003
		Small-fast	3	3.03	0.39
		Control	3	3.22	0.36

Data for hypoxia (10 and 21 kPa po_2_) were analyzed separately from hyperoxia (21, 25, 33, and 40 kPa po_2_). Main effect tests were followed by a priori planned comparisons to determine whether oxygen rearing level had a within-line effect on survival rate.

### Hypoxia effects

With a multivariate analysis that considered all the parametric response variables together, the lines responded similarly to hypoxia (Table 1). Similarly, with univariate analyses that examined responses of growth rate, development time, maximal larval mass, adult mass, and adult length, the three lines responded similarly to hypoxia (no significant interactive effect between atmospheric oxygen level and selection line; Table 2; Figs. 2–6). There was also no significant line × oxygen interactive effect on survival (Table 4). However, survival rate was significantly decreased in hypoxia in the big-fast line, but not in the control or small-fast line (Table 4, Fig. 7), providing some support for the hypothesis that selection for large size and fast development time reduces resistance to hypoxic stress. There were strong and significant negative main effects of hypoxia on most response variables (Tables 4, Figs. 2–7).

### Hyperoxia effects

In contrast to hypoxia, there were interactive effects between oxygen rearing level and selection line in the normoxic to hyperoxic range (21–40 kPa po_2_) in the overall MANOVA (Table 1). Similarly, there were significant interactive effects in many of the univariate analyses. There was a significant interactive effect between selection line and oxygen on growth rate (Table 3). Hyperoxia (25 and 33 kPa) significantly decreased growth rates in the big-fast line, but had no effect on growth rates in the control line, and actually increased growth rates in the 40 kPa small-fast group (Table 3, Fig. 2). Hyperoxia also had a significant interactive effect with selection line on development time (Table 2). Hyperoxia (25 and 33 kPa) significantly increased development time in big-fast line (Fig. 3), while for both the small-fast and the control lines, 25 kPa oxygen increased development time, and the 33 and 40 kPa oxygen reduced development time (Fig. 3). There was no significant interaction between selection line and oxygen for maximal larval mass, adult mass, or length (Table 3). Adult masses and lengths tended to decrease in hyperoxia (Figs. 4–6), but these decreases were only significant in 33 kPa oxygen in the control line.

Hyperoxia differentially affected survival among the lines (Table 4). Hyperoxia significantly reduced survival in the big-fast, but not the control or small-fast lines (Fig. 7).

## Discussion

Many of our results supported our hypothesis that selection for large size and a short development time caused increased susceptibility to oxidative stress in caterpillars. This result may have far reaching implications as the fitness consequences of natural selection showed a tendency to increase size in 79% of studies (Kingsolver and Pfennig 2004), and 84% of studies show that natural selection acts to decrease development time (Kingsolver and Huey 2008). There were strong line × oxygen interactions in responses to hyperoxic treatments likely due to increase oxidative stress, with the big-fast line more negatively affected by hyperoxia than the control or slow-fast line, most strikingly for survival. However, for the parametric factors related to size and growth in the *M. sexta* that survived to adulthood, hyperoxia tended to move the big-fast line to values near to those of the control line, not the lower values that might be expected from reduced stress resistance. Also, in hypoxic stress, the lines reacted similarly. Thus, this study yielded mixed results relative to the hypothesis that selection for large size in a short time leads to phenotypic changes that enhance growth rate at the cost of generalized stress resistance.

### Phenotypic effects of selection in normoxia

As expected, selection for large size in a short time period resulted in *M. sexta* that differed in adult mass and length, with big-fast > control > small-fast (Figs. 5 and 6). The larger size of the big-fast line was achieved primarily by higher growth rates (Fig. 2), though this line also had a longer development time (Fig. 3). The only significant growth-related difference between the small-fast line and the control line was maximal larval mass. Another major effect of selection was a decrease in survival; survival among caterpillars from the big-fast and small-fast line was about half that of the control lines. The mechanism for this effect on survival is not clear. It is not likely that selection eliminated genetic variation as the realized narrow-sense heritabilities for body size and development time were fairly large in the selected lines following 10 generations of selection. It would be useful to examine the effect of such selection on genetic variation, asymmetry, and other measures of animal quality.

What physiological/morphological changes might underlie the higher growth rates observed in the big-fast line? One possibility is that the big-fast caterpillars exhibit higher rates of performance of their eating/digesting/assimilating systems, perhaps due to greater neuronal/hormonal activation of the feeding/digestive systems. The big-fast caterpillars might have greater investment in consumption and assimilation systems (e.g., bigger jaws and guts, more digestive enzymes secreted, more protein synthetic enzymes). If greater investment in these systems occurs, this implies a possible reduced relative investment in systems not related to growth, such as repair and maintenance systems (P450's, DNA repair, heat shock proteins, etc.).

### Effects of hypoxia

In general, there were few line × oxygen interactions within the hypoxia experiment on the parametric factors related to growth and size, indicating that the lines responded similarly to hypoxia (Tables 1, 2, and 4; Figs. 2–6). Hypoxia generally decreased survival and growth rate in *M. sexta*, effects that have been observed in a variety of insects now, including mealworms (Loudon 1988), cockroaches (VandenBrooks et al. 2012), fruit flies (Klok et al. 2009), and grasshoppers (Harrison et al. 2006), as well as a variety of marine invertebrates such as polychaetes (Forbes and Lopez 1990), and vertebrates including several fish (Petersen and Pihl 1995; Chabot and Dutil 1999), turtles (Kam 1993), alligators (Owerkowicz et al. 2009), chickens (Tintu et al. 2007), and humans (Moore et al. 2011). The proximate mechanisms for the hypoxic effects on growth are unclear and likely vary among species. It is plausible that hypoxia limits ATP production later in the instar in *M. sexta*, as the critical po_2_ is very high at this time (Greenlee and Harrison 2005), and metabolic rate does not increase despite mass increase later in the instar (Callier and Nijhout 2011). In the fruit fly, *D. melanogaster*, hypoxia reduces larval and adult size at least partly by reducing feeding rates (Frazier 2007), and may reduce adult size by decreasing cell size (Heinrich et al. 2011).

Hypoxia did not affect development time in *M. sexta*. Similarly, in the mealworm, *Tenebrio molitor*, growth was suppressed without an effect on development time (Loudon 1988). This finding fits with the observation that hypoxic *M. sexta* do not have experimentally demonstrable critical weight (Callier and Nijhout 2011), even though caterpillars molted at different sizes in different oxygen levels. In contrast, hypoxia both slows growth and increases development time in fruit flies (Klok et al. 2009), cockroaches (VandenBrooks et al. 2012), and grasshoppers (Harrison et al. 2006). At present, the reasons for the interspecific variation in the effects of hypoxia on development time are unclear. It may be that these different species have different mechanisms for the control of molting and size. For example, the fact that hypoxia slows growth, extends development, and has no effect on adult size in grasshoppers suggests that these insects have a mechanism to ensure that development is extended until a target size is reached. In contrast, in cockroaches, size, growth, and development rate all decrease in hypoxia, suggesting that hypoxia may affect size-determining mechanisms such as critical weight. Additionally, differences in the tracheal system responses to hypoxia might lead to different tissue po_2_ responses to atmospheric hypoxia, and therefore different developmental responses.

### Effects of hyperoxia

There were strong line × oxygen interactions within the hyperoxia experiment, with the big-fast line generally more strongly affected by hyperoxia (Tables 1, 3, and 4; Figs. 2–7). Survival was strongly suppressed for the big-fast line in 40 kPa po_2_, an effect that was not observed for the control or small-fast lines. We found that *M. sexta* selected for large size/fast growth had size and growth parameters more strongly suppressed by hyperoxia than the control or small-fast lines. In the big-fast line, hyperoxia decreased growth rate, larval and adult size, and increased development time (Figs. 2–6). In contrast, the small/fast and control lines either were unaffected or showed faster growth rates and reduced developmental times in hyperoxia, suggesting that in some cases, benefits to higher oxygen might outweigh negative consequences of reactive oxygen species formation in these lines.

The most striking differences in the lines occurred in growth rates. Hyperoxia suppressed growth rates in the big-fast line, and tended to increase growth rates in the control and small-fast group, with a significant increase for the small-fast line in 40 kPa oxygen (Fig. 2). In normoxia, there was a significant one-way ANOVA line effect, with the big-fast the largest. In contrast, in hyperoxia, growth rate decreased in the big-fast line and increased in the small fast line, resulting in the tendency for small-fast animals to have a higher growth rate at 40 kPa than big-fast animals. For the small-fast lines, it is plausible that hyperoxia might increase growth rates by relieving oxygen limitations on metabolism. The critical po_2_ for *M. sexta* caterpillars rises to near-21 kPa in late-fifth instar caterpillars, suggesting possible oxygen limitation (Greenlee and Harrison 2005); and this possibility is supported by the constant metabolic rate in these caterpillars despite increasing mass after the critical weight (Callier and Nijhout 2011).

There were fewer significant effects of hyperoxia on size, whether larval or adult, than for growth rate or development time, suggesting mechanisms that tend to conserve body size with oxygen-related stress. The significant increase in adult size in the control lines (25 kPa) supports the conclusions of Callier and Nijhout (2011) that mild hyperoxia may allow *M. sexta* to achieve a larger size. Above 33 kPa, the big-fast lines showed significant decreases in size. The control and small-fast lines also tended to decrease in body size above 33 kPa, although only some of these decreases were statistically significant. In general, hyperoxic treatments tended to move the big-fast line toward larval and adult sizes similar to those of the control and small-fast line, suggesting that hyperoxia eliminated the size advantage of the big-fast line.

### Why is the big-fast line more susceptible to hyperoxic stress?

The greater negative response of big-fast line animals to hyperoxia suggests that selection for large size and short development time created caterpillars that were particularly sensitive to oxidative damage, either due to increased production of oxygen radicals or reduced capacity to repair damage caused by oxygen radicals. Possibly, big-fast line animals have higher mass-specific metabolic rates and more mitochondria than small-fast or control animals in order to supply ATP at greater rates to support higher growth rates. If so, this could lead to higher rates of oxygen radical production, since mitochondria are the most important source of oxygen radicals (Turrens 2003).

Big-fast line animals might also have reduced capacities to repair stress-induced damage. As noted above, one possibility is that increased investment in growth machinery is associated with a reduced investment in molecules (e.g., glutathione, superoxide dismutase) that can cope with increased production of reactive oxygen species as is likely to occur in hyperoxia. Big-fast caterpillars might also have reduced levels of molecules that cope with protein unfolding associated with such damage (e.g., heat shock proteins), or molecules that repair damaged DNA or membrane lipids.

Variation in the tracheal system could also lead to the big-fast line being more sensitive to hyperoxia. The higher growth rates of big-fast caterpillars might require a higher tracheal conductance to permit higher levels of metabolism, and that higher tracheal conductance could make these caterpillars more sensitive to hyperoxia (tissue po_2_ might be higher at any given atmospheric po_2_). Another possibility is that the big-fast line animals might be less able to reduce the size of the tracheal system in response to hyperoxia, a hypoxia-inducible factor-mediated process that has been well demonstrated in fruit flies (Jarecki et al. 1999; Henry and Harrison 2004; Centanin et al. 2010). Perhaps selection for faster growth is associated with production of trophic factors (e.g., insulin) that override the normal capacity for the downregulation of tracheal proliferation in response to hyperoxia. If so, the big-fast lines may maintain tracheal systems appropriate for normoxic conditions even in hyperoxia, leading to greater oxygen radical production and oxidative damage.

Our results lend support to the hypothesis that achieving large size in a short period of time leads to costs that impact survival and the capacity to cope with oxidative stress. This is of particular importance as natural selection acts to decrease development time and increase body size. Such trade-offs may explain the large variation in growth rates among populations and species in the absence of predation (Dmitriew 2011). Studies on the biochemical and physiological mechanisms responsible for the trade-offs between high growth rates and stress susceptibility seems likely to reveal important traits for evolutionary theory, agriculture, and medicine.
